# Risk Factors for Perineal Wound Breakdown in Early Postpartum: A Retrospective Case–Control Study

**DOI:** 10.3390/jcm12083036

**Published:** 2023-04-21

**Authors:** Amandine Puissegur, Marie Accoceberry, Marion Rouzaire, Bruno Pereira, Marie Herault, Clément Bruhat, Amélie Delabaere, Denis Gallot

**Affiliations:** 1 Obstetrics and Gynaecology Department, CHU Clermont-Ferrand, 63000 Clermont-Ferrand, France; 2 CIC 1405 CRECHE Unit, INSERM, CHU Clermont-Ferrand, 63000 Clermont-Ferrand, France; 3 Biostatistics Unit (DRCI), CHU Clermont-Ferrand, 63000 Clermont-Ferrand, France; 4 Translational Approach to Epithelial Injury and Repair Team, GReD, CNRS, INSERM, Auvergne University, 63000 Clermont-Ferrand, France

**Keywords:** perineal tear, lacerations, case–control studies, risk factors

## Abstract

We conducted a retrospective case–control study in patients presenting a perineal tear (second degree or higher) or episiotomy complicated by wound breakdown during maternity stay to identify risk factors associated with wound breakdown in early postpartum with a view to improving the quality of care. We collected ante- and intrapartum characteristics and outcomes at the postpartum visit. In all, 84 cases and 249 control subjects were included. In univariate analysis, primiparity, absence of history of vaginal delivery, a longer second phase of labour, instrumental delivery, and a higher degree of laceration emerged as risk factors for early perineal suture breakdown postpartum. Gestational diabetes, peripartum fever, streptococcus B, and suture technique did not emerge as risk factors for perineal breakdown. Multivariate analysis confirmed that instrumental delivery (OR = 2.18 [1.07; 4.41], *p* = 0.03) and a longer second phase of labour (OR = 1.72 [1.23; 2.42], *p* = 0.001) were risk factors for early perineal suture breakdown.

## 1. Introduction

Perineal wound breakdown following childbirth is a rare complication, but strongly impacts women’s quality of life. Traditionally managed expectantly, it can take several weeks for the wound to fully heal. It can require additional care, including surgical revision [1,2,3]. It can delay return home and impugn the quality of care provided. It can cause persistent pain, discomfort at the perineal wound site, infection, vaginal bleeding, urinary retention, defaecation problems, and dyspareunia [4,5,6]. 

The exact incidence of disunion is not known but it is generally between 0.1% and 2.1%, depending on the degree of the initial tear. It reaches 24.6% in patients with an initial fourth-degree tear.

Many studies have been published on the risk factors for perineal tears [7,8,9,10,11,12], but there are relatively few on the risk factors for wound breakdown postpartum owing to the rarity of this complication. The risk factors for perineal wound breakdown described in the literature to date are nulliparity, smoking, episiotomy, operative delivery (especially forceps [13]), third- and fourth-degree laceration, wound repair by a midwife, infection, use of chromic sutures, BMI > 35 kg/m^2^, estimated blood loss > 500 mL and postpartum narcotic use [6,13,14,15,16,17]. By contrast, the use of a prophylactic dose of intravenous amoxicillin and clavulanic acid after operative vaginal delivery has been shown to reduce the risk of perineal wound breakdown [18].

Early perineal wound breakdown raises the question of how the surgical procedure is best performed. Considering that early disunion is not necessarily linked to the patient’s condition, this study was designed to identify factors linked to the obstetrical context and to the performance of the procedure independently of the patient’s own healing capacity.

Some risk factors such as age [19], BMI and smoking [20] are widely known to reduce healing capacities. Besides these factors that are directly related to the patient’s condition, early breakdown may be more strongly influenced by the obstetrical context, including the course of delivery, severity of perineal trauma, perineal repair—techniques of suture, operator, and early complications—haematoma or clinical infection (fever). Moreover, complications in early postpartum are known to cause maternal dissatisfaction. In this context, our objective was to identify the risk factors for perineal wound breakdown in early postpartum with a view to improving the quality of care.

## 2. Materials and Methods

### 2.1. Study Design and Inclusion Criteria

We carried out a retrospective case–control study including all patients who gave birth in the maternity ward of the Estaing University Hospital in Clermont-Ferrand from 1 January 2010 to 30 April 2015 and who presented a perineal wound breakdown of a tear of the 2nd degree or higher or an episiotomy in early postpartum (i.e., diagnosed during their stay in the maternity ward).

These patients were found in the Auvergne ICOS database, which contains all the files of patients who gave birth at the Estaing Hospital during the period studied.

Wound breakdown was defined as the separation of one or more stitches from the perineal suture, whatever its size, depth or location.

The standard classification of perineal tears was used [21].

− 1st degree: superficial injury to the vaginal mucosa that may involve the perineal skin.
− 2nd degree: tear of the skin, vaginal mucous membrane and perineal muscles
− 3rd degree: tear of the skin, vaginal mucosa, perineal muscles and anal sphincter
− 4th degree: tear of the skin, vaginal mucosa, perineal muscles, anal sphincter and rectal mucosa.

### 2.2. Variables

The different variables studied were:

− Antepartum variables: age, ethnicity, socio-economic status, tobacco use (before and during pregnancy), BMI, parity, history of vaginal delivery, history of vaginal tear or episiotomy.
− Intrapartum variables: gestational diabetes, carrying streptococcus B, maternal temperature at admission and delivery, antibiotic therapy during labour, presence of meconium fluid during labour, duration of rupture of membranes, duration of second stage of labour, episiotomy, obstetrical manoeuvre (vacuum, forceps, Jacquemier’s manoeuvre or manoeuvre for breech presentation), type of suture (single running suture or conventional three-stage technique), child’s birth weight.
− Postpartum variables: maternal fever, haematoma, and perineal oedema.
− At the post-natal visit: residual pain, loss of substance, bridles, resumption of intercourse.

### 2.3. Statistical Analysis

With the objective of the study being to identify the risk factors for perineal wound breakdown suture disunion in early postpartum, sample size was estimated by a rule of thumb, counting the number of patients concerned relative to the number of possible predictors of perineal wound breakdown. The ratio of three controls to one case was used to ensure satisfactory 90% statistical power. 

Control subjects were randomly selected from a list of patients who had a vaginal tear or episiotomy without breakdown in the same period and matched for age, BMI and tobacco use during pregnancy, all established factors in wound healing defects.

All statistical analyses were performed with Stata software (version 13, StataCorp, College Station, TX, USA). A difference was considered statistically significant when the significance level was less than 0.05 (two-sided type I error at 5%). 

Categorical variables were described by numbers and associated percentages. Continuous variables were expressed as mean (and standard deviation) or median [interquartile range] according to their statistical distribution. The assumption of distribution normality was tested using the Shapiro–Wilk test. Comparisons between groups were performed using Student’s *t*-test or the Mann–Whitney test if the conditions to apply the *t*-test were not met. Homoscedasticity was analysed by the Fisher–Snedecor test. The comparisons between groups for categorical variables were made with the chi-squared test or where appropriate with Fisher’s exact test. For multivariate analyses, logistic regression was then performed with covariates determined with regard to the univariate results and their clinical relevance. A directed acyclic graph (DAG) was proposed by three specialists (two obstetricians and one midwife) to minimise multicollinearity (Supplementary Materials). The results were expressed with odds ratios (OR) and 95% confidence intervals.

## 3. Results

**Figure 1:**
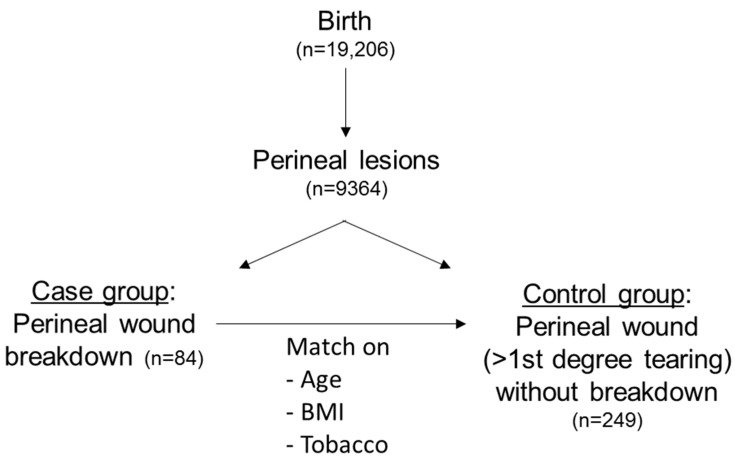
Flow chart of the study.

There were 19,206 births between January 2010 and May 2015, including 15,319 births by vaginal delivery with 9364 perineal lesions over the study period (episiotomies and perineal lesions of the first degree or higher) (Figure 1). 

In all, 84 cases and 249 controls were included in the study according to the inclusion criteria. The mean age and body mass index of women enrolled in the study were 29.2 ± 5.3 and 23.1 ± 4.3, respectively. The proportion of smokers among the participants was 19.8%. 

In the wound breakdown group, there were 23 breakdowns at the introitus vaginal fourchette, 7 skin breakdowns, 49 limited superficial vaginal breakdowns and 5 total suture breakdowns. Of the patients who suffered a wound breakdown, four required revision surgery. The remaining patients were treated locally until healing, with weekly supervision by a midwife at home or in our unit for the most serious breakdowns.

The characteristics of cases and controls were similar except for parity, with statistically more primiparous women in the case group (83.3% vs. 69.9% *p* = 0.02) and fewer patients with a history of vaginal birth in the case group (10.90% vs. 24.10% *p* = 0.02) (Table 1).

The intrapartum factors differed over the duration of the second stage of labour, with a median of 65 min [13.5–130] in cases compared with 25 min [7–80] in controls (*p* = 0.001). Patients in the case group had an earlier gestational age (39.5 ± 1.6 weeks) than controls (40.0 ± 1.1 weeks, *p* = 0.03). The degree of tearing was greater in patients with breakdown than in those without (*p* = 0.02). There was a statistically significant difference in the performance of an obstetrical manoeuvre, with 47.6% of patients in the case group versus 27.7% in the control group (*p* = 0.001) with more vacuum (39.3% in the case group versus 22.5% in the control group *p* = 0.003) and more forceps at the time of expulsion (8.3% in the case group versus 1.6% in the control group *p* = 0.007) (Table 2).

The factors monitored at the maternity ward were similar for both groups (Table 3).

Gestational diabetes, peripartum fever, streptococcus B, and suture technique did not reach significance. The postpartum visit conducted between 6 and 8 weeks postpartum was analysed for 168 women (48 case and 120 control). There was a statistically higher prevalence of substance loss and presence of bridles in the case group and earlier resumption of intercourse in the control group (Table 4).

Multivariate analysis confirmed that an instrumental delivery (OR = 2.18 [1.07; 4.41], *p* = 0.03) and a longer second phase of labour (OR = 1.72 [1.23; 2.42], *p* = 0.001) were risk factors for early wound breakdown (Table 5).

## 4. Discussion

Perineal wound breakdown is rare, but the unpredictability of this complication prompted us to seek predisposing factors, especially related to the obstetrical context, and so identify a profile of patients with a higher risk of early breakdown with a view to improving the quality of care. Compared with existing literature reports, the originality of our study was to focus on factors independent of the healing capacity of the patients. 

Our study showed a rate of wound breakdown of 0.9%, in line with findings in similar retrospective studies [6,14,22].

In agreement with the literature, primiparity [14] and the absence of a history of vaginal delivery [6] were found to be predisposing factors in our study. Likewise for the degree of tearing and instrumental delivery, which have been found to be risk factors in most studies evaluating scar breakdown [6,13,14,22,23].

Consistent with several other studies, no difference between different suture techniques was found [24,25]. There was more wound breakdown when the suture was performed by a resident or a physician, which probably indicated a recruitment bias, since these persons suture the most extensive tears and those following an instrumental delivery, which are at greatest risk of wound breakdown.

In the postpartum period, less resumption of intercourse and more residual anatomical lesions suggest an impact on women’s experience and an adverse outcome for their sexual health and subsequent pregnancies. Nevertheless, the proportion of missing data was high for the postpartum visit (50% of the women were not followed up at the hospital for the postpartum visit) and patient responses may be biased, as the mode of investigation was not by self-questionnaires but by responses reported directly to the physician. Although the therapeutic management of wound breakdown was not one of the criteria of our study, a very low rate of surgical revision was noted. In a review of the literature carried out in July 2013 based on the Cochrane Database [2], a non-significant difference in healing at 4 weeks was shown between the failures resurfaced surgically and those treated by local care. Similarly, the rate of dyspareunia was identical between the two groups. In contrast, more women in the secondary suture group had resumed sexual intercourse at two months (RR 1.78, 95% CI 1.10–2.89, one study, thirty-five women), although at six months there was no significant difference between the two groups (RR 1.08, 95% CI 0.91–1.28). A qualitative study published in 2017 looked at the personal experience of women with perineal wound breakdown and their views on the proposed management. The testimonies confirmed the extent of the morbidity felt, while revealing a strong preference for the option of revision surgery over an expectant attitude [3].

Multivariate analysis showed that risk factors for early wound breakdown were variables specific to the delivery (instrumental delivery and duration of the second stage of labour). Primiparity, first vaginal delivery or perineal haematoma did not reach significance in the multivariate model. 

As a longer duration of second stage of labour is also known to be a risk factor of post-partum haemorrhage [26], these patients could experience both complications (i.e., early postpartum haemorrhage and early wound breakdown).

Our study did not identify gestational diabetes, peripartum fever or carrying streptococcus B as risk factors for breakdown. 

Williams and Chames [6] found a 40.7% rate of infection documented in their retrospective study of 59 cases between 1995 and 2005. Several other studies report that infection of the episiotomy suture was the principal source of wound breakdown [27,28,29,30]. Predisposing factors were contamination at the time of suturing, the presence of necrotic tissue, poor perineal hygiene and the formation of a haematoma.

In our study, we did not report any severe maternal sepsis and our clinical practice did not include any additional investigation to characterize a possible infection.

We did not find any difference in the presence of oedema, haematoma or meconial amniotic fluid, the appearance of which might point to later wound breakdown. However, these variables were subjectively assessed by caregivers, so may be biased. 

The main limitation of our study was to include only early perineal wound breakdown, because women were included until hospital discharge. It is possible that wound breakdown occurred after maternity ward discharge in patients who did not return to the hospital for medical care. Nevertheless, complications in early postpartum are known to contribute to maternal dissatisfaction and we can hypothesise that focusing on the early postpartum period enabled us to study factors related to the obstetrical context (including course of delivery, severity of perineal trauma, perineal repair and early complications) rather than those linked to the patient’s condition.

## 5. Conclusions

By focusing on variables independent of the inherent healing capacity of the patients, we were able to identify predisposing factors especially related to the obstetrical context with a view to improving the quality of care.

Primiparity, the absence of a history of vaginal delivery, a longer second phase of labour, instrumental delivery and a greater degree of tearing were risk factors for early perineal wound breakdown postpartum. Multivariate analysis confirmed that instrumental delivery and a longer second phase of labour were risk factors.

## Figures and Tables

**Table 1:** Patient characteristics.

	Case N = 84	Control N = 249	OR (95% CI)	*p*-Value
**Ethnic origin**				0.82
Europe,	49 (82%)	88 (83%)	Reference	
Maghreb,	7 (12%)	10 (9.5%)	2.19 (0.88–5.43)	0.09
Africa,	0	1 (0.95%)	0.22 (0.03–1.75)	0.15
Asia,	3 (5%)	3 (2.8%)	0.79 (0.22–2.94)	0.73
Other	1 (1.6%)	3 (2.8%)	0.37 (0.04–2.98)	0.35
**Low socio-economic status**	14 (16.7%)	37 (14.9%)	0.90 (0.46–1.76)	0.76
**Primiparous**	70 (83.3%)	174 (69.9%)	2.15 (1.14–4.06)	0.02
**History of vaginal delivery**	10 (10.90%)	60 (24.10%)	0.42 (0.20–0.87)	0.02
**History of episiotomy or vaginal tear**	9 (10.71%)	40 (16.06%)	0.63 (0.29–1.35)	0.23

**Table 2:** Intrapartum characteristics.

	Case *N* = 84	Control *N* = 249	OR (95% CI)	*p*
**Gestational diabetes**	12 (14.3%)	19 (7.6%)	2.02 (0.93–4.35)	0.07
**Streptoccocus B carriers**	11 (13.1%)	30 (12.2%)	1.08 (0.52–2.27)	0.8
**Temperature at admission (°C)**	37.0 ± 0.4	37.0 ± 0.3	0.91 (0.41–2.01)	0.13
**Temperature at delivery (°C)**	37.4 ± 0.5	37.3 ± 0.5	1.76 (0.85–3.63)	0.07
**Antibiotic during labour**	13 (15.5%)	53 (21.3%)	0.68 (0.35–1.32)	0.25
**Membrane rupture time (hours)**	6 [4–15]	6 [3–11]	1.01 (0.99–1.03)	0.053
**Duration of the 2nd phase (minutes)**	65 [13.5–130]	25 [7–80]	1.67 (1.27–2.20)	0.001
**Gestational age (weeks)**	39.7 ± 1.5	39.9 ± 1.2 SA	0.91 (0.75–1.09)	0.37
**Meconial amniotic fluid**	15 (17.9%)	64 (25.7%)	0.63 (0.34–1.17)	0.14
**Episiotomy**	60 (71.4%)	151 (60.6%)	1.62 (0.95–2.77)	0.08
without tearing	57 (95%)	141 (93.3%)		
with 2nd degree tearing	1 (1.6%)	4 (2.6%)		
with 3rd degree tearing	2 (3.3%)	6 (4.0%)		
**Degree of tearing**				
2nd degree	20 (74.1%)	99 (91.7%)	Reference	
3rd degree	6 (22.2%)	9 (8.3%)	3.30 (1.05–10.31)	0.02
4th degree	1 (3.7%)	0	Not estimated	
**Obstetrical manoeuvre**	40 (47.6%)	69 (27.7%)	2.37 (1.42–3.95)	0.001
Vacuum	33 (39.3%)	56 (22.5%)	2.23 (1.31–3.78)	0.003
Forceps	7 (8.3%)	4 (1.6%)	5.57 (1.58–19.52)	0.01
Other	3 (3.6%)	9 (3.6%)	0.98 (0.26–3.74)	0.64
**Birth weight (g)**	3288 ± 462	3295 ± 418	0.99 (0.99–2.60)	0.9
**Suture in three planes**	59 (70.2%)	168 (67.5%)	1.13 (0.66–1.95)	0.64
**Running suture Operator**	25 (29.8%)	81 (32.5%)	0.88 (0.51–1.50)	
Resident	35 (41.6)	80 (32.1)	Reference	
Physician	10 (11.9)	14 (5.6)	1.63 (0.66–4.02)	0.64
Midwife	39 (46.4)	155 (62.2)	0.57 (0.34–0.98)	0.02

**Table 3:** Patient characteristics at the maternity ward.

	Case *N* = 84	Control *N* = 249	OR (95% CI)	*p*
**Fever**	1 (1.2%)	3 (1.2%)	1.12 (0.11–10.96)	1
**Perineal haematoma**	9 (10.7%)	16 (6.4%)	2.03 (0.85–4.87)	0.1
**Oedema**	6 (7.1%)	23 (9.2%)	0.86 (0.33–2.23)	0.1
**Missing data**	22 (26.2%)	40 (16.1%)		

**Table 4:** Characteristics during the postnatal visit.

	Case *N* = 48	Control *N* = 120	OR (95% CI)	*p*
**Pain**	4 (8.3%)	19 (16.2%)	0.47 (0.15–1.45)	0.18
Missing	0	4		
**Loss of substance**	6 (12.5%)	1 (0.8%)	16.99 (1.99–145.36)	0.002
Missing	0	0		
**Bridles**	9 (18.7%)	4 (3.3%)	6.69 (1.95–22.95)	0.002
Missing	0	0		
**Resumption of intercourse**	9 (18.7%)	49 (40.8%)	0.34 (0.15–0.77)	0.005
Missing	1	0		

**Table 5:** Multivariate analysis.

	OR (95% CI)	*p*
**Primiparous**	1.87 (0.38–9.25)	0.44
**History of vaginal delivery**	1.60 (0.26–9.76)	0.61
**Duration of the 2nd phase (minutes)**	1.72 (1.23–2.42)	0.001
**Instrumental delivery (vacuum or forceps)**	2.18 (1.07–4.41)	0.03
**Perineal haematoma**	1.83 (0.66–5.04)	0.24

## Data Availability

The data presented in this study are available on request from the corresponding author.

## Supplementary Materials

The following supporting information can be downloaded at: https://www.mdpi.com/article/10.3390/jcm12083036/s1, File S1: The directed acyclic graph (DAG) for the study. The shaded variables were the most frequently found among the authors.
