# Supplementary material for: Risk Factors for Perineal Wound Breakdown in Early Postpartum: A Retrospective Case–Control Study
Source: J Clin Med. 2023 Apr 21;12(8):3036. doi: 10.3390/jcm12083036 (PMC10146046; doi:10.3390/jcm12083036)

Supplementary File S1 : The directed acyclic graph (DAG) for the study.

The shaded variables were the most frequently found among the authors.

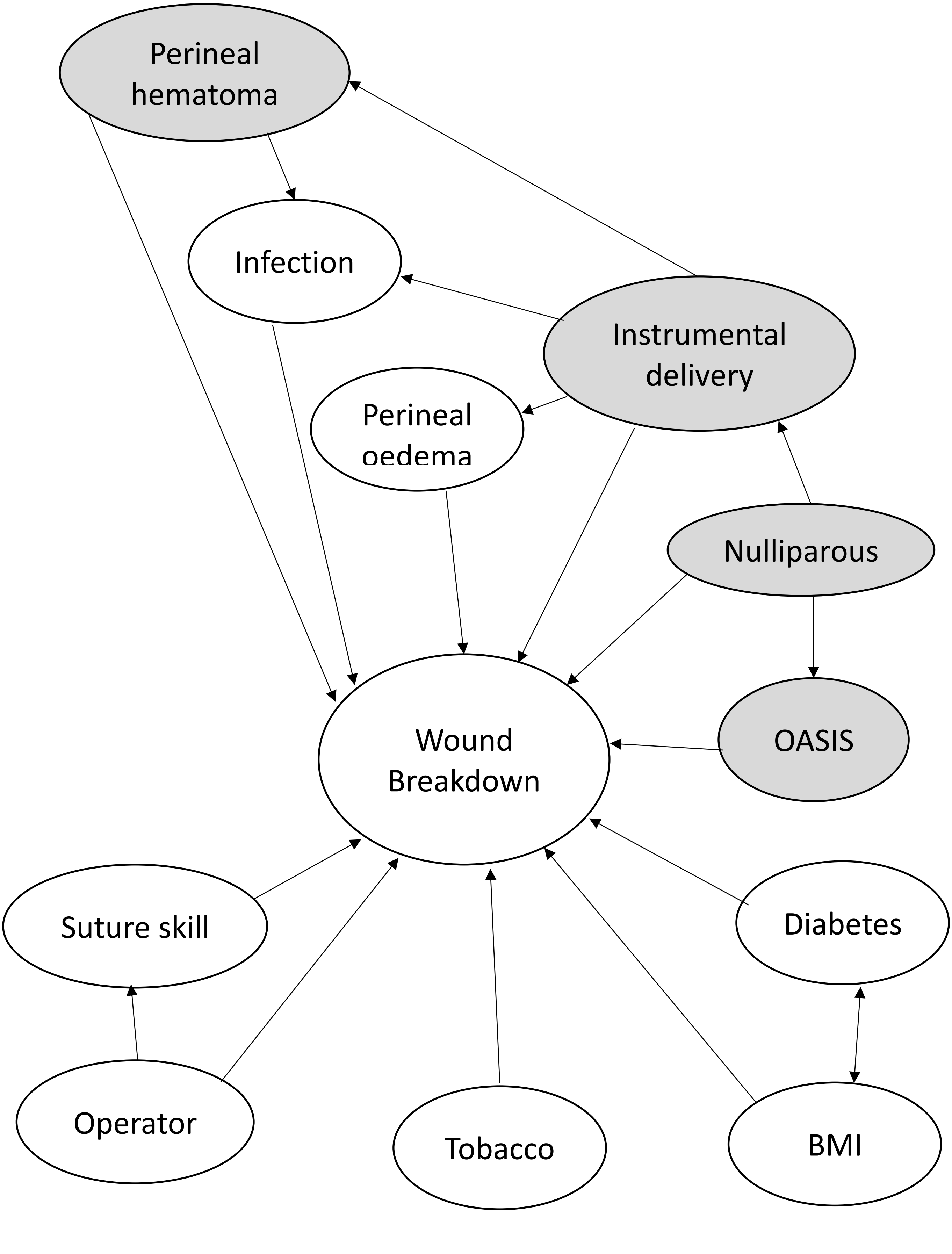

Supplement: Supplementary file 1 [file jcm-12-03036-s001.zip › jcm-2303409-supplementary.pdf]
